# FGF-2 Deficiency Does Not Influence FGF Ligand and Receptor Expression during Development of the Nigrostriatal System

**DOI:** 10.1371/journal.pone.0023564

**Published:** 2011-08-18

**Authors:** Andreas Ratzka, Olga Baron, Claudia Grothe

**Affiliations:** 1 Institute of Neuroanatomy, Hannover Medical School, Hannover, Germany; 2 Center for Systems Neuroscience (ZSN), Hannover, Germany; Consejo Superior de Investigaciones Cientificas, Spain

## Abstract

Secreted proteins of the fibroblast growth factor (FGF) family play important roles during development of various organ systems. A detailed knowledge of their temporal and spatial expression profiles, especially of closely related FGF family members, are essential to further identification of specific functions in distinct tissues. In the central nervous system dopaminergic neurons of the substantia nigra and their axonal projections into the striatum progressively degenerate in Parkinson's disease. In contrast, FGF-2 deficient mice display increased numbers of dopaminergic neurons. In this study, we determined the expression profiles of all 22 *FGF-ligands* and 10 *FGF-receptor* isoforms, in order to clarify, if FGF-2 deficiency leads to compensatory up-regulation of other FGFs in the nigrostriatal system. Three tissues, ventral mesencephalon (VM), striatum (STR) and as reference tissue spinal cord (SC) of wild-type and FGF-2 deficient mice at four developmental stages E14.5, P0, P28, and adult were comparatively analyzed by quantitative RT-PCR. As no differences between the genotypes were observed, a compensatory up-regulation can be excluded. Moreover, this analysis revealed that the majority of FGF-ligands (18/22) and FGF-receptors (9/10) are expressed during normal development of the nigrostriatal system and identified dynamic changes for some family members. By comparing relative expression level changes to SC reference tissue, general alterations in all 3 tissues, such as increased expression of *FGF-1*, *-2*, *-22*, *FgfR-2c*, *-3c* and decreased expression of *FGF-13* during postnatal development were identified. Further, specific changes affecting only one tissue, such as increased *FGF-16* (STR) or decreased *FGF-17* (VM) expression, or two tissues, such as decreased expression of *FGF-8* (VM, STR) and *FGF-15* (SC, VM) were found. Moreover, 3 developmentally down-regulated FGFs (FGF-8b, FGF-15, FGF-17a) were functionally characterized by plasmid-based over-expression in dissociated E11.5 VM cell cultures, however, such a continuous exposure had no influence on the yield of dopaminergic neurons *in vitro*.

## Introduction

Fibroblast growth factor 2 (FGF-2) is a member of the FGF family, which comprises small proteins of about 150–300 amino acids length with a common conserved core domain [1]. FGF-2, like several other secreted FGFs, is involved in distinct processes during development of the central nervous system (CNS) and possess neurotrophic properties for a wide range of mature neurons [2]–[5]. In particular, FGF signaling regulates patterning processes in different brain areas [6]–[8], proliferation of neuronal progenitor cells and neuronal differentiation [9]–[12]. FGFs are involved in formation of functional neural networks by regulating axonal outgrowth, synapse formation and specification [13]–[15]. Moreover, FGF-2 has physiological relevance for dopaminergic (DA) neurons of the nigrostriatal system [16] and FGF-2 depletion might be related to Parkinson's disease [17].

Based on their mode of action, the 22 mammalian FGFs have been classified into intracrine, canonical and hormone-like FGFs [1], [18], [19]. The intracrine FGFs (FGF-11/12/13/14), also known as FGF homologous factors (FHFs, Table 1), interact with intracellular domains of voltage gated sodium channels and are involved in modifying the electrical excitability of neurons [20], [21]. In contrast, canonical FGFs are secreted proteins, which function in an autocrine/paracrine manner. They form ternary complexes with heparan sulfates and extracellular domains of transmembrane FGF-receptors (FgfRs). Formation of such complexes initiates receptor dimerization followed by autophosphorylation of the intracellular FgfR tyrosine kinase domain and subsequent signal transduction. Based on phylogenetic analysis, the canonical FGFs can be subdivided into 5 subfamilies: FGF-1/2/5, FGF-3/4/6, FGF-8/17/18, FGF-7/10/22 and FGF-9/16/20 [1]. The remaining FGF ligands belong to the hormone-like (endocrine) FGFs (FGF-15/19/21/23) thereof FGF-15 (mouse) and FGF-19 (human) are orthologous genes. Hormone-like FGFs possess a much lower binding affinity to FgfRs and heparan sulfates compared to canonical FGFs, which is balanced upon binding of the respective co-receptors α-Klotho or β-Klotho [19].

**Table 1:** Overview of *FGF-ligand* and *FGF-receptor* expression during CNS development.

	qRT-PCR data			ALLEN brain atlas ISH data		
Gene	expression level	developmental expression	Figure	SC	VM	STR
**FGF receptors**						
FgfR-1b	low	stable	S1A	- *	- *	- *
FgfR-1c	high	stable	1B	E11-P56 *	E11-P56 *	P4-P56 *
FgfR-2b	n.d. - low	stable	S1B	- *	- *	- *
FgfR-2c	moderate - high	up	1D	E11-P56 *	E11-P28 *	P14-P28 *
FgfR-3b	low	stable	S1C	- *	- *	- *
FgfR-3c	moderate - high	up	1E	E11-P56 *	E11-P28 *	P14-P28 *
FgfR-4	low	down/stable	S1D	n.d.	n.d.	n.d.
FgfRl1	moderate	up/stable	1C	P4, P56	P14	n.d.
α-Klotho	moderate	stable	1A	P4, P56	-	-
β-Klotho	n.d. – low	-	-	P4, P56	-	-
**canonical FGF ligands (grouped into subfamilies)**						
**FGF-1/2**						
FGF-1	low - high	up	2A	P4, P56	E11-P56	P4-56
FGF-2	low - moderate	up	2B	n.d.	n.d.	n.d.
**FGF-4/5/6**						
FGF-4	n.d. - low	-	-	P56	n.d.	n.d.
FGF-5	low	stable	S1E	P56	n.d.	n.d.
FGF-6	n.d.	-	-	n.d.	n.d.	n.d.
**FGF-3/7/10/22**						
FGF-3	low - moderate	up/stable	2D	P4, P56	E13-E15	n.d.
FGF-7	moderate	stable	S1F	n.d.	n.d.	n.d.
FGF-10	low - moderate	stable	2E	n.d.	n.d.	n.d.
FGF-22	low - moderate	up	2C	-	-	-
**FGF-8/17/18**						
FGF-8	n.d. - low	down	2J	n.d.	E11, E13	n.d.
FGF-17	low - moderate	down/stable	2I	n.d.	n.d.	n.d.
FGF-18	moderate	down/stable	2K	P4, P56	E18-P56	P4
**FGF-9/16/20**						
FGF-9	moderate	stable	S1G	E11,P4,P56	E11,P4-P28	P4–P28
FGF-16	n.d. - moderate	up/stable	2F	n.d.	n.d.	n.d.
FGF-20	n.d. - low	stable	2L	-	P4–P28	P4–P28
**intracrine FGF ligands**						
FGF-11	moderate	stable	S1H	P4, P56	-	-
FGF-12	high	stable	S1I	P4, P56	P4-P56	P4-P56
FGF-13	high	down	2G	-	-	-
FGF-14	moderate - high	stable	S1J	E15,P4,P56	E15-P28	P4-P28
**hormone-like FGF ligands**						
FGF-15	n.d. - moderate	down	2H	E11-E15,P4	E18	n.d.
FGF-21	n.d.	-	-	n.d.	-	-
FGF-23	n.d.	-	-	-	n.d.	n.d.

Quantitative RT-PCR expression levels were classified based on ΔC_T_ values to *Gapdh* reference gene into: high (ΔC_T_<6), moderate (ΔC_T_ 6–11), low (ΔC_T_>11–15) or not detected (n.d., ΔC_T_>15). The qRT-PCR data is summarized across all tissues VM, STR and SC and all developmental stages analyzed, for detailed expression profiles see indicated figures. Available ISH datasets of the ALLEN brain atlas were analyzed for up to 8 stages (E11.5, E13.5, E15.5, E18.5, P4, P14, P28, P56) for SC, VM and STR. The table summarizes developmental stages with detectable expression.

*Although, *FgfR-1*, *FgfR-2* and *FgfR-3 in situ* probes are homologous to FgfR c spliceforms, also b splice forms might be detected. Due to high abundance of c isoforms in CNS most likely these isoforms have been detected by ISH.

Mammals possess four different FGF-receptors (FgfR-1,-2,-3,-4) of which FgfR-1, -2 and -3 occur in different isoforms, which originate through alternative splicing. The two major signal transducing b and c FgfR isoforms differ in their third extracellular Ig-like domain, which confers FGF ligand specificity. Different binding preferences of individual FGFs for different FgfR and receptor isoforms have been identified [22], [23]. Moreover, the complexity of FGF-FgfR interactions may be further increased by the ability of FgfR to form heterodimers [22]. In addition, another FGF receptor FgfR-like1 (FgfRl1 or FgfR-5) displays similarities to extracellular ligand binding domains of the canonical FgfRs but lacks the intracellular kinase domain. Therefore, FgfRl1 likely acts as a decoy receptor sequestering FGFs away from canonical FgfRs [24].

Mice deficient for individual FGF-ligands display phenotypes ranging from mild to early embryonic lethal [1]. Likewise, rather small phenotypic differences in the CNS of FGF-2 deficient mice have been identified, such as reduced numbers of astrocytes in the hindbrain and reduced numbers of specific neuron subtypes in the cerebral cortex, hippocampal formation and spinal cord [12], [25], [26]. The specification of a more severe phenotype might be prevented by functional redundancy of co-expressed FGF-ligands. However, no synergistic phenotypes have been observed in either FGF-2/FGF-1 or FGF-2/FGF-5 double-deficient mice [27], [28]. Our recent morphometric analysis of the nigrostriatal system revealed, as an additional phenotype of FGF-2 deficient mice, an increased number of substantia nigra DA neurons [29]. Based on their binding affinity to FgfR-3c and presence in the VM, several other FGFs have been proposed as candidates, which might compensate for FGF-2 deficiency in the nigrostriatal system [16]. However, to date only fragmentary information on the expression profiles of particular FGFs in the nigrostriatal system are available. To fill this gap, we comprehensively analyzed the expression of all 22 *FGF-ligands* and 10 *FGF-receptors* (Table 1) by quantitative RT-PCR (qRT-PCR). Particular focus was laid on the comparison of wild-type and FGF-2 deficient mice in order to identify a possible compensatory up-regulation of other FGF family members due to FGF-2 deficiency. Our analysis of the nigrostriatal system, i.e. ventral mesencephalon (VM) and striatum (STR), and as a reference tissue spinal cord (SC), in four developmental stages embryonic (E14.5), newborn (P0), juvenile (P28) and adult (AD) mice, revealed that FGF-2 deficiency did not affect expression of any other FGF-ligand or FGF-receptor. Moreover, unique insights on the dynamic changes of the expression levels of individual FGFs were obtained. Based on this analysis three in the VM developmentally down-regulated FGF-ligands (FGF-8b, FGF-15 and FGF-17a) were selected and their effect on DA neuron differentiation was studied after over-expression in a well established *in vitro* assay.

## Results

### FGF-2 deficiency does not affect expression levels of other FGF ligands and receptors

*FGF-ligand* and *FGF-receptor* expression was analyzed for 11 separate cDNA samples, comprised of 3 tissues and 4 developmental stages, from both wild-type and FGF-2 deficient mice. During the first analysis of pooled cDNA samples (see methods) small differences between both genotypes ranging from ΔC_T_ values of 0.3 to 1.0 were identified for some genes. However, differences did not reach statistical significance (p>0.05) after the subsequent analysis of individual cDNA samples (n = 3–7, data not shown). While loss of FGF-2 apparently had no effect on the expression of other FGF-ligands or receptors, at least on the transcriptional level, our analysis identified both developmentally regulated and stably expressed genes (see below).

### The FGF-system in the developing nigrostriatal system

**Figure 1:**
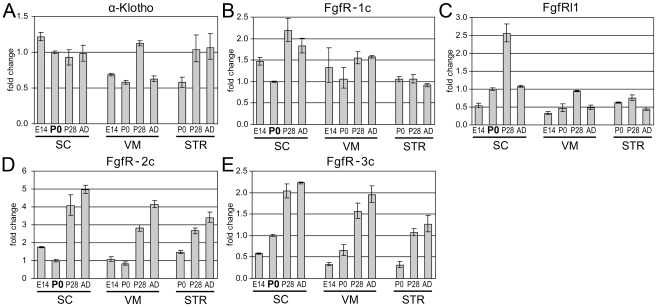
Expression profiles of the major *FGF-receptors*. (**A,B**) Expression of *α-Klotho* (A) and *FgfR-1c* (B) remained stable throughout development of SC, VM and STR. (**C**) *FgfRl1* was temporary up-regulated 5 fold in P28 SC and 3 fold in P28 VM, but remained stable in STR. (**D,E**) Expression of *FgfR-2c* (D) and *FgfR-3c* (E) increased in all three tissues in a range of 3–6 fold from E14.5 to AD stage, except SC *FgfR-2c* expression increased only 2.3 fold. Note the different scaling of the y-axis.

Given the complexity of the FGF-system, our analysis of three CNS regions (VM, STR, SC) revealed that the majority of the *FGF*-ligands (18 out of 22) and *FGF-*receptors (9 out of 10) are expressed in at least two, in most cases throughout all developmental stages analyzed (Table 1). Exceptions included *FGF-6*, *FGF-21* and *FGF-23*, which were not detected in any tissue analyzed, and *FGF-4* and *β-Klotho*, which were absent in most tissues except of low levels of *FGF-4* (ΔC_T_ = 14.8) and *β-Klotho* (ΔC_T_ = 13.5) in adult SC and P28 SC, respectively. To discriminate between abundant and rare transcripts, the ΔC_T_ value, which is calculated by subtracting the C_T_ value of the highly expressed *Gapdh* reference gene from the C_T_ value of the gene of interest, was used to define four expression level categories: high (ΔC_T_<6), moderate (ΔC_T_ = 6 to 11), low (ΔC_T_>11 to 15) or not detected (ΔC_T_>15) (Table 1, Table S1). To allow a better comparison of the expression profiles of individual genes, expression levels were normalized to P0 SC, which was set to 1 (except for *FGF-20* to P0 VM and *FgfR-2b* to E14.5 SC, which were both not detected in P0 SC). The comparison of five *FGF-receptors*, which displayed moderate and high expression levels, identified stable expressed or developmentally up-regulated genes (Fig. 1). Levels of *α-Klotho* and *FgfR-1c* remained stable during development of SC, VM and STR (<2 fold changes, Fig. 1A,B), whereas expression of *FgfRl1* remained stable in the STR but was temporary up-regulated at P28 in VM (3 fold) and SC (5 fold) (Fig. 1C). Expression levels of *FgfR-2c* and *FgfR-3c* increased from E14 to AD, most prominently after birth, in all tissues examined, mostly in a range of 3–6 fold, except for STR *FgfR-2c* 2.3 fold (Fig. 1D,E). The remaining *FGF-receptor* isoforms (*FgfR-1b*, *-2b*, *-3b* and *-4*) displayed low expression levels and were mostly stable expressed (<2 fold changes) during all developmental stages and tissues examined, except for up-regulation of *FgfR-1b* in STR and *FgfR-3b* in VM, and down-regulation of *FgfR-4* in SC (Fig. S1A–D).

**Figure 2:**
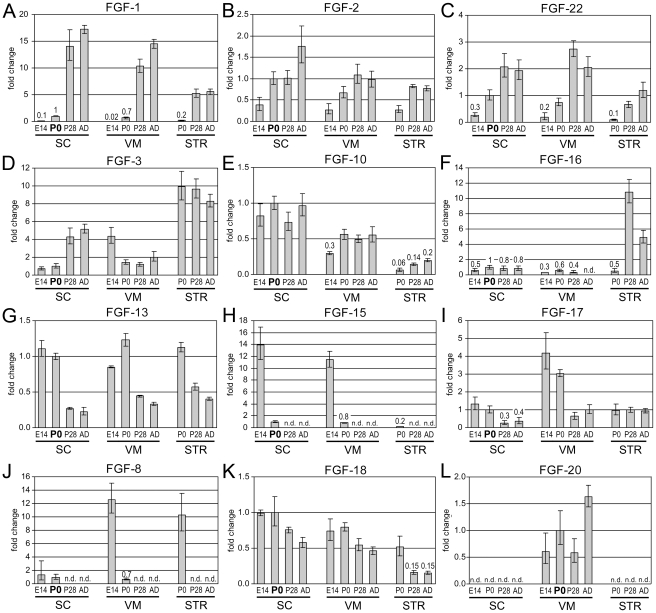
Differentially expressed *FGF-ligands*. (**A–F**) Six *FGF-ligands* were up-regulated (>3 fold) during development, either in all three tissues, such as *FGF-1* (A), *FGF-2* (B) and *FGF-22* (C), or in single tissues, such as *FGF-3* in SC (D), *FGF-10* in STR (E) and *FGF-16* in STR (F). (**G–K**) Five *FGF-ligands* were down-regulated (>3 fold) during development, *FGF-13* in SC (G), *FGF-15* (H) and *FGF-17* (I) both in SC and VM (H), *FGF-8* in VM and STR (J) and *FGF-18* in STR (K). (**L**) Expression of *FGF-20* was detected specifically in the VM at all stages. Note the different scaling of the y-axis.

Eleven *FGF-ligands* appeared in at least one tissue to be developmentally regulated, displaying >3 fold changes between E14 (or P0 for STR) and the AD stage (Fig. 2A–I). *FGF-1*, *-2*, *-22* were up-regulated during development in all three tissues (Fig. 2A–C), whereas expression of *FGF-3*, *-10*, *-16* increased only in SC or STR, respectively (Fig. 2D–F). *FGF-13* expression decreased during postnatal stages in all three tissues, with a 5 fold decrease in SC and to a lesser extend in VM and STR (2.6–2.8 fold) (Fig. 2G). Expression of *FGF-15* and *FGF-17* decreased in SC and VM (Fig. 2H,I), and *FGF-8* decreased in VM and STR (Fig. 2J). *FGF-18* was 3 fold down-regulated in the STR (Fig. 2K). In addition to differences seen during development of an individual tissue, some genes displayed differences between CNS tissue types. Expression of *FGF-1*, *-10*, *-15*, *-18* was highest in SC and VM (Fig. 2A,E,H), whereas expression of *FGF-3* and *FGF-16* was highest in STR (Fig. 2D,F). Strongest expression in the VM was observed for FGF-17 and FGF-20, while FGF-8 expression was highest in E14.5 VM and P0 STR (Fig. 2I,J,L). The remaining *FGF-ligands FGF-5*, *-7*, *-9*, *-11*, *-12*, *-14* displayed stable expression levels (<2 fold changes) throughout all stages and tissues (Fig. S1E–J). The classification of moderately and highly expressed FGF-ligands into FGF subfamilies, revealed that all four intracrine FGFs were highly expressed at all stages, whereas only *FGF-15* among the hormone-like FGFs was moderately expressed in E14.5 SC and VM (Table 1). All canonical FGF subfamilies, with the exception of the FGF-4/5/6 subfamily, contained members, which were expressed at moderate to high levels (Table 1). It is interesting to note, that individual *FGF-ligands* were either up-regulated, stable or down-regulated during development, whereas down-regulation of moderately or highly expressed *FgfRs* was never observed, indicating that FGF signaling was maintained by various FGF-ligands during development.

### Comparison with ALLEN Brain Atlas ISH database

The quantitative RT-PCR expression data was compared with the publicly accessible *in situ* hybridization (ISH) databases of the Allen Institute for Brain Science [30], which comprises sagittal sections of whole mouse embryos (E11.5–E15.5) or brains (E18.5–P56) (http://developingmouse.brain-map.org/) and SC cross sections (P4 and P56) (http://mousespinal.brain-map.org/). High resolution bright field ISH pictures and false colorized ‘expression mask’ pictures were available for most *FGF receptors* and *FGF ligands* (except *FGF-11*, *-13*, *-21*, *-22*, *α-Klotho*, *β-*Klotho in the brain database, and *FGF-13*, *-20*, *-22*, *-23* in the SC database, as determined on April 2011). ISH expression levels of *FGF-receptor* and *FGF-ligands* have been summarized in Table 1. In agreement with moderate to high qRT-PCR expression levels of *FgfR-1*, *FgfR-2* and *FgfR-*3 all three FgfRs have been detected at various stages in the ALLEN ISH databases, whereas the moderately expressed *FgfRl1* was detected only in few postnatal stages SC (P4, P56) and VM P14. Expression of *FgfR-4* was not detected by ISH, which was in agreement with the low expression level seen by qRT-PCR. Of note, although *FgfR-1*, *FgfR-2* and *FgfR-3* ISH riboprobes were identical to the respective c isoform, they contained also homologous regions to the respective b isoform. As the c isoforms are more abundantly expressed in the CNS (Table 1), the reported ISH expression pattern correspond most likely to this isoform. Furthermore, distinction of low or absent gene expression was difficult for some transcripts, for example brightfield pictures of *α-Klotho* and *β-Klotho* at P56 SC, displayed both lightly stained cells scattered throughout the gray matter, whereas false colorized ‘expression mask’ pictures were devoid of *β-Klotho* expressing cells but contained few blue and green colorized cells for *α-Klotho*. In agreement to that, qRT-PCR analysis revealed moderate levels of *α-Klotho* and low levels or absent expression of *β-Klotho* at P28 and adult SC, respectively.

Notably, most FGFs which had been classified by qRT-PCR as highly or moderately expressed were also detectable by ISH, whereas low level expressed genes were not detected by ISH (Table 1), which most likely reflects different sensitivities of both methods. In the SC cells expressing *FGF-1*, *-3*, *-9 -11*, *-12*, *-14*, *-1*8 were scattered throughout the gray matter in P4 and P56 SC. In addition, *FGFs-1*, *-9*, *-11*, *-18* displayed an increased expression domain in the ventral horn of the SC. In contrast *FGF-15* expression was restricted to the roof plate of the SC at E11.5–E15.5 and to few cells close to the central canal at P4. *FGF-ligands* which displayed robust ISH expression in the SC were mostly also detected in VM and STR, however, some exceptions such as *FGF-3* were noted (Table 1). *FGF-3* expression in the VM (E11.5–E15.5) was regionally restricted, similar as seen for *FGF-15* in the VM at E18.5. This expression domain did not overlap with the substantia nigra and most likely corresponds to the interstitial nucleus of cajal, for which *FGF-15* expression has been previously described at E16.5 and P7 [31]. Furthermore, *FGF-15* was strongly expressed in the dorsal mesencephalon from E11.5 to E18.5. *FGF-8* was expressed at the midbrain-hindbrain border at E11.5 and E13.5. In agreement with qRT-PCR data, *FGF-18* ISH expression in the STR decreased from P4 to P14, whereas expression in the VM remained stable. Expression of *FGF-14* was detected throughout the brain from E15.5–P28, while *FGF-12* expression was detected only in postnatal stages (P4–P28). The two remaining intracrine *FGFs* (*FGF-11*, *-13*) have not been incorporated in the ALLEN brain ISH database. *FGF-1* was ubiquitously expressed in the brain from E11.5 onwards and additionally at postnatal stages regionally more intense especially in the brain stem. In some cases staining of P56 stage was weaker compared to E11-P28 stages, which might reflect different ISH riboprobes used for both ISH datasets included in the ALLEN brain atlas. For example *FGF-20* was ubiquitously expressed in the brain at P4 and P28 stages, but was not detected at P56.

### Over-expression of selected FGF-ligands

**Figure 3:**
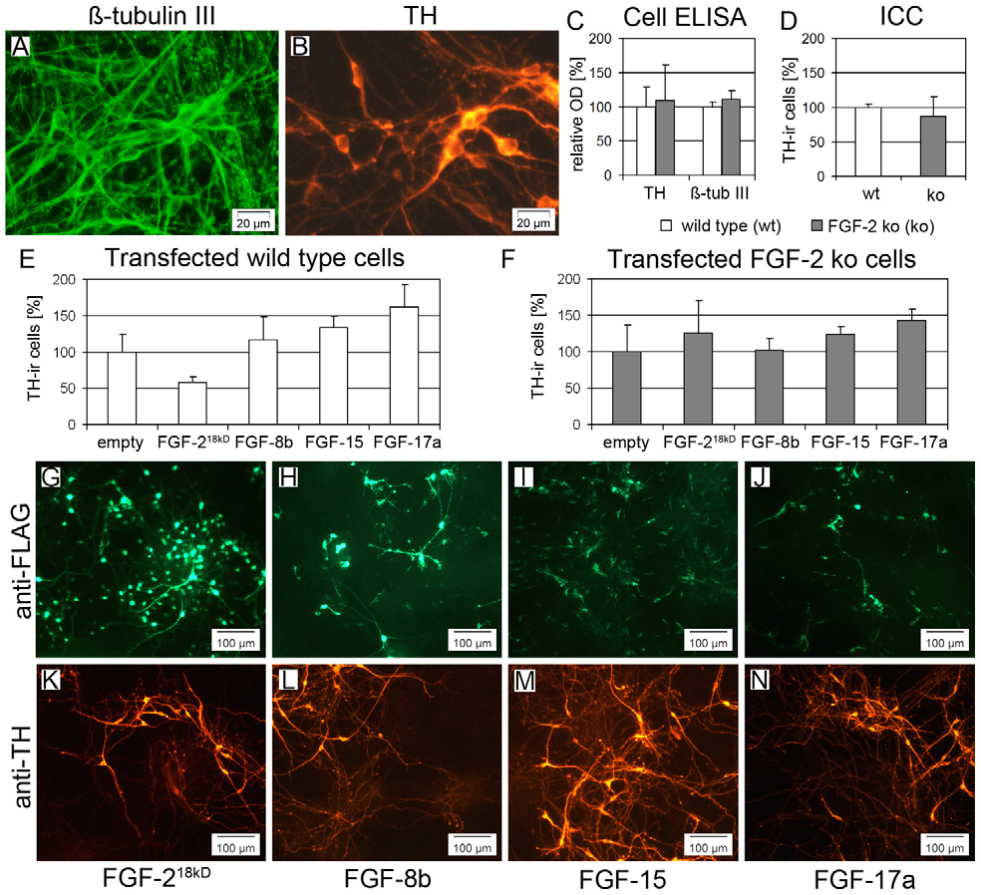
Over-expression of FGF-2^18kDa^, FGF-8b, FGF-15 or FGF-17a does not influence differentiation of DA neurons *in vitro*. (**A–D**) Comparative evaluation of E11.5 derived VM cultures from wild-type and FGF-2 deficient mice revealed similar numbers of neurons (ß-tubulin III-ir, A) and DA neurons (TH-ir, B), quantified either by cell-ELISA (C) or immuncytochemistry (D). (**E–N**) The transient over-expression of FGF-2^18kDa^ (G,K), FGF-8b (H;L), FGF-15 (I,M) or FGF-17a (J,N), did not significantly increase the yield of TH-ir cells (K-N) neither in wild-type (E) nor in FGF-2 deficient VM cells (F).

As the substantia nigra of adult FGF-2 deficient mice contains more DA neurons compared to wild-type animals [29], we were interested if such differences could be observed during *in vitro* differentiation of DA progenitor cells. Therefore, E11.5 VM cells from either wild-type or FGF-2 deficient mice were cultured for 6 days under differentiation condition *in vitro*. However, comparative evaluation of tyrosine hydroxylase immunoreactivity (TH-ir), which is the rate-limiting enzyme of DA biosynthesis, revealed no significant difference between the genotypes by cell ELISA technique for neuronal marker ß-Tubulin III (Fig. 3A) and TH (Fig. 3B, Fig. 3C). While the results for the ß-Tubulin III-ir cell ELISA measurements remained stable across the experiments, the results for TH-ir measurement varied, probably due to subtle differences in the age and maturation stage of DA precursor cells of the dissected brains. In addition, counting of TH-ir cells revealed similar numbers of DA neurons of FGF-2 deficient and wild-type derived cells confirming the cell ELISA data (Fig. 3D).

Since *FGF-8*, *FGF-15* and *FGF-17* showed high expression levels specifically in the embryonic VM and were down-regulated during development, we analyzed whether a continuously high availability of these FGFs affects TH-ir cell differentiation *in vitro*. Therefore, FGF expression plasmids encoding either for FGF-2^18kDa^, FGF-8b, FGF-15 or FGF-17a were transiently transfected in primary cultures of E11.5 VM cells, derived either from FGF-2 deficient mice or wild type mice. Cells transfected with empty-plasmid served as control and were set to 100%. Immunocytochemical detection of transfected cells by targeting the FLAG-epitope revealed approximate 10–20% FLAG-ir cells 6 days after transfection (Fig. 3G–J). Quantification of TH-ir cell numbers of FGF- and empty-plasmid transfected controls revealed no significant differences neither in wild-type nor FGF-2 deficient VM cell preparations (Fig. 3E–F). In addition, semi quantitative examination of three independent transfection experiments, revealed no obvious differences between wild-type and or FGF-2 deficient cells (data not shown).

## Discussion

As we previously identified a new phenotype of FGF-2 deficient mice of increased numbers of DA neurons in the adult substantia nigra [29], we found in the present study that a compensatory up-regulation of other *FGF-ligands* or *FGF-receptors*, at least on the transcript level, does not take place in the nigrostriatal system or SC of FGF-2 deficient mice. Moreover, by using the sensitive qRT-PCR technique most *FGF-ligands* (18/22) and *FGF-receptors* (9/10) could be detected in VM or STR samples, although abundance of individual genes differed strongly. Grouping of individual FGF-ligands by FGF subfamilies revealed that all four intracrine FGFs are abundantly expressed, whereas hormone-like FGFs are mostly not expressed, except for expression of *FGF-15* in early stages. Particularly interesting for a potential role during DA neuron differentiation and maintenance are secreted canonical and hormone-like FGFs expressed in the VM. Three types of expression profiles were discriminated during VM development, up-regulated (*FGF-1*, *-2*, *-22*), down-regulated (*FGF-8*, *-15*, *-17*) or constantly expressed (*FGF-5*, *-7*, *-9*, *-10*, *-16*, *-18*, *-20*). The comparison of nigrostriatal expression levels to an unrelated region, the SC, revealed that most developmental changes applied to all three CNS areas, indicating that the observed developmental changes reflect general aspects of CNS development. On the other hand, the few tissue specific distinctions at a given stage, such as increased expression of *FGF-3* and *FGF-16* in the STR, *FGF-17* and *FGF-20* in the VM, and *FGF-8* in VM and STR might point to tissue specific roles of these genes. Indeed, *FGF-20* has been identified to be expressed in the substantia nigra and shown to enhance the survival of DA neurons [32], [33]. Further, mutations in the *FGF-20* gene locus have been associated with an increased risk for Parkinson's disease [34]. Early embryonic expression of *FGF-8* and *FGF-17* in the midbrain-hindbrain boundary has been shown to be important for correct patterning of the brain and proper development of the midbrain [8], [35]–[37]. In early mouse embryos *FGF-3* expression has been detected in the midbrain and the lateral ganglionic eminence (precursor of the striatum) of the telencephalon, [38], [39]. However, the impact of *FGF-3* and *FGF-16* expression in postnatal striatum on maturation or maintenance of DA neurons has to our knowledge not been studied.

Distinct ligand specificities of FgfRs have been identified via mitogenic assays of transfected BaF3 cells in comparison to FGF-1, which was used as internal control since it could activate all FGFR isoforms [22], [23]. For example, FGF-8 and -17, which were down-regulated during VM development, activated FgfR3c more strongly than FgfR1c and 2c, of which FgfR2c and 3c were up-regulated during VM development, whereas FgfR1c remained stable. The hormone-like FGF-15, which was down-regulated as well, displayed only weak activity for all canonical FgfRs. As the ß-klotho co-receptor of FGF-15 [19] was not detectable in the developing VM, an improved FGF-15/FgfRs interaction can be excluded. The developmentally up-regulated FGF-2 activated FgfR1c and 3c more strongly then FgfR-2c. In contrast, FGF-22 activated FgfR-2b and -1b more pronounced, which are expressed at low levels in the VM, however weak activation of FgfR-1c, which is expressed at high levels in the VM, could be an alternative. Although those *in vitro* ligand specificities provide a base line for comparison of different FGFs, their binding specificities *in vivo* are modified by varying sulfation patterns of heparan sulfate co-receptors present in distinct tissues and developmental stages [40], [41].

### FGFs and DA neuron development

FGF-2 is a mitogen for VM neuronal precursor cells and suppresses their differentiation into DA neurons *in vitro* [11]. Application of either FGF-2 or FGF-8 recombinant protein into cell cultures of dissociated VM cells have been shown to increase proliferation and to result, after subsequent FGF withdrawal, in increased numbers of DA neurons compared to FGF untreated controls [42]–[44]. The situation *in vivo* appears more complex, as FGF-2 deficient mice display increased numbers of DA neurons [29]. However, in this study we showed that dissociated cell cultures derived either from E11.5 wild-type or FGF-2 deficient mice, respectively, generate similar numbers of DA neurons *in vitro*, which might indicate that differentiation regulating factors are missing in such cultures. Secreted FGF-ligands, which are known to influence neuron differentiation, are excellent candidates to fulfill such function. In particular, *FGF-8b* expression at the midbrain-hindbrain boundary (MHB) is required for midbrain specification (including the substantia nigra) and has been shown to regulate rostrally directed growth of DA axons [4], [14]. Other isoforms belonging to this FGF subfamily, such as FGF-8a, FGF-17b and FGF-18, have been shown to possess distinct properties for midbrain and hindbrain patterning, compared to FGF-8b [36], [37], whereas another member FGF-17a has not been analyzed so far. FGF-15 promotes neural differentiation in the dorsal mesencephalon and frontal cortex [9], [10]. Furthermore, *FGF-8*, *FGF-15* and *FGF-17* were expressed in E14.5 VM and down-regulated during subsequent developmental stages as determined by qRT-PCR. However, over-expression of FGF-8b, FGF-15, FGF-17a or FGF-2^18kDa^ *in vitro* had no influence on the number of mature DA neurons neither in wild-type nor in FGF-2 deficient VM cell cultures, indicating that other factors and/or mechanism lead to the increased DA neuron numbers seen in FGF-2 deficient mice *in vivo*. One possible explanation for the multifunctionality of FGF-2 might be attributed to different isoforms expressed in cells, whereas for *in vitro* experiments only 18 kDa FGF-2 was applied. The FGF-2 transcript contains up-stream CUG translation initiation codons, which yields high molecular weight (HMW) FGF-2 isoforms of 21 and 23 kDa, in addition to the AUG codon which forms the canonical 18 kDa FGF-2 isoform. HMW FGF-2 is exclusively localized in the nucleus, while 18 kDa FGF-2 can be found in the nucleus, cytoplasm and extracellularly released. Moreover, distinct effects of 18 kDa FGF-2 and HMW FGF-2 isoforms have been identified, such as regulation of gene expression, protein interaction with SMN and nuclear FgfR1/CBP/RSK1 complexes [45]–[49].

### FGF expression studies

Diverse techniques, such as immunohistochemistry, northern blot, qRT-PCR, non-radioactive and radioactive ISH, which are characterized by different assets and drawbacks in terms of sensitivity, throughput and spatial information, have been applied to analyze expression of FGF-ligands and FGF-receptors. Using immunohistochemical methods FGF-1 and FGF-2 have been detected in DA neurons of the adult substantia nigra [50]. Further, expression during development was monitored by northern blot and revealed constant expression levels for *FGF-2* from E16.5 to adulthood, whereas *FGF-1* expression was not detected before P20 and increased further to P90 [51]. Similarly, our qRT-PCR analysis for the developing VM revealed a rather small 3 fold up-regulation of *FGF-2* and a striking 80 fold up-regulation of *FGF-1* between E14.5 and AD stage. In addition to the high detection sensitivity, qRT-PCR technique offers the possibility to discriminate between individual splice forms, such as b and c *FgfR* isoforms, which differ by one alternatively spliced exon. Further, qRT-PCR expression profiles of individual genes can be compared, to identify similarly expressed FGF family members, for example during development of a particular tissue, like the developing nigrostriatal system (this study), or across diverse organ systems at a particular developmental stage [52]. The two independent FGF and FgfR primer sets of both studies are a valuable tool to address similar questions in the future.

Complementary information on cellular and tissue-wide expression patterns can be provided by ISH technique, like the ALLEN brain atlas ISH database [30], which confirmed the expression of highly expressed and some of the moderately expressed genes from our qRT-PCR expression study. Differences observed between the ALLEN brain atlas (enhanced non-radioactive ISH) and radioactive ISH studies performed by others, might reflect different sensitivities of both methods or design of the ISH riboprobes. For example, in agreement with our qRT-PCR analysis, expression patterns for all 4 intracrine FGFs have been identified by radioactive ISH in the murine CNS at E12.5, E18.5 and adult stages [53], whereas *FGF-12* was not detected at embryonic stages in the ALLEN brain atlas. Further, expression of *FGF-20* has been confined to substantia nigra by radioactive ISH [32], which is in agreement to low levels of *FGF-20* detected by qRT-PCR specifically in the VM, whereas the ALLEN brain atlas reports ISH data for two distinct riboprobes, which displayed *FGF-20* either as ubiquitously expressed (P4–P28) or as not detected (P56).

### Conclusion

Whereas FGF-2 deficiency leads to increased numbers of nigral DA neurons, expression levels of other *FGFs* and *FgfRs* were not altered in the nigrostriatal system, which excludes a compensatory up-regulation of the FGF-system at least on the transcriptional level. However, it is still possible that the numerous *FGFs* expressed (at normal levels) in the CNS are sufficient to prevent the appearance of a more severe phenotype of FGF-2 deficient mice. Future studies on compound mutant mice deficient for FGF-2 and other FGFs, which are also expressed in the embryonic VM, such as different members of the FGF-8/17/18 subfamily, might uncover synergistic effects on DA neuron differentiation.

## Materials and Methods

### FGF-2 deficient mice and tissue processing

The FGF-2 deficient mice strain (FGF-2^tm1Zllr^) was maintained on C57BL/6 background [25]. This mutation replaces the 1^st^ exon of FGF-2 with a neomycin expression cassette, thereby CUG and AUG start-codons of high and low molecular weight FGF-2 isoforms were removed. Wild-type (FGF-2^+/+^, wt) and FGF-2 deficient (FGF-2^−/−^, ko) littermates were obtained by crossbreeding of heterozygous FGF-2 mice. For time pregnancies, noon on the day of the vaginal plug was defined as embryonic (E) day 0.5. Genotyping was performed by PCR using improved primers, FGF-2_GT2_wtF: 5′-CTCCTGGCCTTAACCCTTTCT-3′, FGF-2_GT2_wtR: 5′-GAGGGATCAAGTCAGGCTTTG-3′ and FGF-2_GT_NeoR: 5′-CCCGTGATATTGCTGAAGAGC-3′. PCR conditions were 95°C for 30 sec, 58°C for 30 sec, and 72°C for 60 sec for a total of 31 cycles, which generated PCR products of 470 bp and 820 bp for the wild-type and mutant allele, respectively. All experimental protocols followed German law on animal care and were approved by Bezirksregierung Hannover, Germany (33.9-42502-04-08/1487).

### Quantitative RT-PCR

Immediately after cervical dislocation, VM, STR and SC were dissected from of FGF-2^+/+^ and FGF-2^−/−^ littermates. Tissue samples were snap frozen in liquid nitrogen and stored at −80°C. Animals were genotyped by PCR (see above) from genomic DNA isolated from tail tissue samples. RNA was extracted from tissue samples of individual animals for P28 and adult stages, respectively. For E14.5 and P0 stage, respectively, two individual tissue samples were combined each, due to the small tissue size. Tissue was homogenized in Trizol reagent (Invitrogen) and total RNA was extracted as recommended by the manufacturer. To eliminate any genomic DNA contamination a DNase (Stratagene) digest was performed. Total RNA (1 µg) was converted into cDNA using the iScript cDNA synthesis kit including a blend of oligo(dT) and random hexamers (BioRad). For initial screening experiments aliquots of individual cDNA samples isolated either from FGF-2^+/+^ or FGF-2^−/−^ genotype were pooled. The pooled cDNA contained for E14.5 and P0 stage 3 individual cDNA samples per genotype (except 2 for P0 STR), for P28 stage 4 individual cDNA samples per genotype and for adult stage 4 FGF-2^+/+^ and 5 FGF-2^−/−^ cDNA samples. In case the ΔΔC_T_ values of FGF-2^+/+^ and FGF-2^−/−^ pooled cDNA samples differed by more then 0.3 cycles, qRT-PCR was repeated with individual samples (n = 3–7), which included fresh aliquots of the original cDNAs (used also for cDNA pooling) and if necessary samples from additional animals. The primers for *glyceraldehyde-3-phosphate dehydrogenase* (*Gapdh*) have been adapted from [54]. All other primer sequences were designed with primer3 software and spanned exon-intron boundaries. Expected size and melting points of the PCR-products are included in Table S1. To verify the correct size of the PCR products, qRT-PCR reactions were exemplarily separated on a 2% agarose gel (Fig. S2). The qRT-PCR was performed in 96-well plates using the StepOnePlus instrument (Applied Biosystems) as described previously [55]. After qRT-PCR cycling, dissociation curves were calculated for each well and melting points were compared to the values reported in Table S1, to ensure specificity of the PCR product. Equal PCR efficiency of most primer pairs (except for very low expressed FGF-20, FGF-21, FGF-23) were validated by serial cDNA dilutions of CNS cDNA samples or alternative tissue sources (whole E12.5 embryos, whole brain or muscle tissue).

The data was analyzed with the StepOne™ software version 2.1 (Applied Biosystems), with a constant threshold value of 0.2. Fold changes in mRNA levels compared to wild type littermates were calculated using the method and normalized to the housekeeping gene *Gapdh*. Two additional housekeeping genes: *hypoxanthine guanine phosphoribosyl transferase* (*Hprt*) and *peptidylprolyl isomerase A* (*Ppia*) were tested, which displayed small differences in few samples compared to *Gapdh*. In particular VM expression of *Hprt* was reduced to 0.4 fold at E14 and increased to 1.4 fold at P28 (Fig. S1K), whereas expression of *Ppia* was decreased to 0.5 fold in all three tissues at postnatal stages (Fig. S1L). Expression levels below the detection limit, with ΔC_T_ values above 15 or which yielded no PCR product at all were assigned as not detected (n.d.). Raw ΔC_T_ values for P0 SC are included in the Table S1. Although, we used ΔC_T_ values for a crude classification into highly, moderately or lowly expressed genes (Table 1), it has to be noted that a more detailed comparison of the expression levels of different genes is generally not possible, due to the fact that conversion of mRNA transcripts into cDNA occurs with different efficiencies for individual genes. However, the relative quantification method used allows a accurate comparison of gene expression levels of a given gene across different cDNA samples, such as tissue types and stages, in relation to the SC P0 reference tissue used for normalization. Expression levels in other stages and tissues are depicted as fold changes compared to this reference tissue, which was set to 1 (indicated by bold type set on the x-axis of Fig. 1, Fig. 2, Fig. S1).

### DA neuron cell culture and transfection

Dissection of mouse E11.5 VM and preparation of the dissociated cell cultures was performed as previously described for rat E12.5 VM cultures [44]. 30.000–40.000 cells/well were seeded on polyornithine coated 96-well plates in attachment medium, which contained 3% FCS (PAA), 20 ng/ml FGF-2 (Preprotech), 1× B27 (Gibco), 1× N2 (Gibco), for 1 day. Differentiation of DA neurons was initiated by culturing for 6 days in differentiation medium, which contained 1× B27, 1% FCS and 100 mM ascorbic acid, but no N2 or FGF-2. Detailed composition of the DMEM/F12 (Gibco) based media have been published previously [44]. Cells used for transfection were cultured after the first day in attachement medium for 2 additional days in proliferation medium, which resembled the composition of adhesion medium omitting FCS and B27 supplement. On DIV 3 transfection was performed using 0.5 µl Lipofectamine 2000 reagent (Invitrogen) and 0.2 µg plasmid DNA per well as recommended by the manufacture. After transfection cells were incubated for 4–6 hours in proliferation medium, followed by 6 days in differentiation medium. Expression plasmids were derived from pCAGGS plasmid, which contained the CAG-promoter, kindly provided by Dr. Hitoshi Niwa, RIKEN Center for Developmental Biology, Japan [56]. Cloning of the c-terminal 3xFLAG tagged enhanced green fluorescence protein (EGFP) expression plasmid pCAGGS-EGFP-FLAG (R412) has been described previously [57]. The coding sequence of 18 kDa rat FGF-2 (NM_019305.2, 533–994 bp), rat FGF-8b (corresponds to rat FGF-8a NM_133286.1, 1–612 bp, with an 33 bp insertion of GTAACTGTTCAGTCCTCACCTAATTTTACACAG between 69 and 70 bp), rat FGF-15 (NM_130753.1, 1–654 bp) and rat FGF-17a (corresponds to rat FGF-17b NM_019198.1, 1–648, without 33 bp CAGGGGGAGAATCACCCGTCTCCTAATTTTAAC between 69–103 bp) was amplified by PCR from rat E12 embryonic cDNA (PCR primer sequences are available upon request). *Eco*RI- or *Mfe*I-sites followed by a kozak sequence were introduced by the forward primer and the stop-codon was replaced by *Xba*I-site by the reverse primer, which allowed in frame cloning to the 3xFLAG tag of the *Eco*RI/*Xba*I digested pCAGGS-FLAG plasmid backbone. Thereby, the FGF expression plasmids pCAGGS-FGF2-18kDa-FLAG (R417), pCAGGS-FGF8b-FLAG (R421), pCAGGS-FGF15-FLAG (R423) and pCAGGS-FGF17a-FLAG (R424) were generated.

### Immuncytochemistry, cell ELISA and cell counting

For fluorescence immunocytochemistry cells were fixed with 4% PFA in PBS for 20 min at room temperature and blocked with PBS containing 0.3% Triton X-100, 5% normal goat serum (NGS) and 1% BSA for one hour at room temperature. The primary antibodies rabbit anti-TH (1∶500, Chemicon AB-152), mouse anti-FLAG M2 (1∶250, Sigma F-1804), mouse anti-β-Tubulin III (1∶250, Upstate Biotech, 05-559) were diluted in PBS containing 0.3% Triton X-100, 1% NGS and 1% BSA and incubated overnight at 4°C. The fluorochrome-conjugated secondary antibodies (Invitrogen, A11001, A21429, A11008) were applied for 1 hour. For staining of cell nuclei 4′,6-diamidino-2-phenylindole (DAPI) was applied in a dilution of 1∶1000 in PBS for 5 min. Photographs were taken with AnalySIS software (Olympus) on an inverted microscope (Olympus, IX70) supplied with a UV lamp and Olympus ColorView 2 camera. TH-ir cell number was quantified with ImageJ on 5 images (4× objective) or 9 images (10× objective) per well, in 3 wells per independent experiment. Quantification of TH-ir cell number of non-transfected E11.5 VM derived cultures was repeated twice. Transfection experiments were performed four times, of which TH-ir cell number was semi quantitatively examined in 3 experiments, whereas the 4^th^ representative experiment was quantified (3 wells per plasmid).

Cell enzyme-linked immunosorbent assay (cell ELISA) of primary E11.5 VM cultures was performed in 96-well plates as previously described in 4 independent experiments with 3–7 replicates per group [58]. Minor modifications included, fixation of the cells in methanol at −20°C for 10 min, cell permeabilization and blocking was performed in PBS containing 10% horse serum, 1% NGS and 0.3% Triton X-100. The primary monoclonal antibodies, mouse anti-TH (1∶200, Chemicon, MAB 5280), mouse anti-ß-Tubulin III (1∶140, Upstate Biotech, 05-559) were applied in PBS containing 1% NGS and 0.3% Triton X-100 overnight at 4°C. Bound primary antibodies were detected by peroxidase-based avidin-biotin complex (Elite ABC kit, Vectastain) and 2,2′-azino-bis(3-ethylbenzthiazoline-6-sulphonic acid) (ABTS, Vectastain) was used as peroxidase substrate. The relative absorbance was measured at 405 nm with microplate reader ELX800 (Bio-Tek Instruments, Inc). Data was corrected for unspecific staining in control wells omitting the first antibody.

### Statistical analysis

The qRT-PCR data are expressed as means ± SD. Statistically significance of the individual cDNA samples between genotypes were determined with unpaired Student's t-test. The *in vitro* data are expressed as means ± SEM. TH-ir cell numbers of the differently transfected groups were tested by Kruskal-Wallis-test (including Dunns post hoc test) using GraphPad Prism 4 software. P-values below 0.05 were considered as statistically significant.

## Supporting Information

Figure S1 **Low expressed** ***FgfR-receptors*** **, stable expressed** ***FGF-ligands*** **and additional control genes.** (**A–D**) Expression of the low expressed *FgfR-1b* (A), *FgfR-2b* (B), *FgfR-3b* (C) and *FgfR-4* (D) remained stable (<2 fold changes) throughout development of most tissues analyzed, except for 3 fold increased *FgfR-1b* in STR (A), 3 fold increased *FgfR-3b* in VM (C) and temporary decreased expression of *FgfR-4* in all 3 tissues at P28 (D). (**E–J**) Six *FGF-ligands FGF-5* (E), *FGF-7* (F), *FGF-9* (G), *FGF-11* (H), *FGF-12* (I), *FGF-14* (J) remained stable expressed throughout all stages and tissues analyzed. (**K,L**) Two additional control genes *Hprt* (K) and *Ppia* (L) showed minor variation (between 0.3 to 1.4 fold changes) compared to *Gapdh* used for normalization. Expression of *Ppia* was consistently 2 fold decreased in stages P28 and AD in all three tissues. Note the different scaling of the y-axis.
(TIF)

Figure S2 **PCR product gel analysis.** After qRT-PCR cycling, PCR reactions were separated on a 2% agarose gel together with a 100 bp size marker (100 bp–1 kb in 100 bp steps). All primer pairs produced single PCR-products of the expected size (compare Table S1).
(TIF)

Table S1 **FGF and FgfR primer sequences.** Characteristic parameters of qPCR-products (length and melting point) are summarized in the 3^rd^ column. Raw ΔC_T_ values obtained from the reference tissue (P0 SC or exceptions AD SC, P0 VM, E14 SC, P28 SC) are indicated in the 4^th^ column. Abbreviations: aFGF, acidic FGF; bFGF, basic FGF; C_T_, threshold cycle; FHF, fibroblast growth factor homologous factor; SC, spinal cord; STR, striatum; VM, ventral mesencephalon.
(DOC)
