# Supplementary figures and images for: FGF-2 Deficiency Does Not Influence FGF Ligand and Receptor Expression during Development of the Nigrostriatal System
Source: PLoS One. 2011 Aug 18;6(8):e23564. doi: 10.1371/journal.pone.0023564 (PMC3158085; doi:10.1371/journal.pone.0023564)

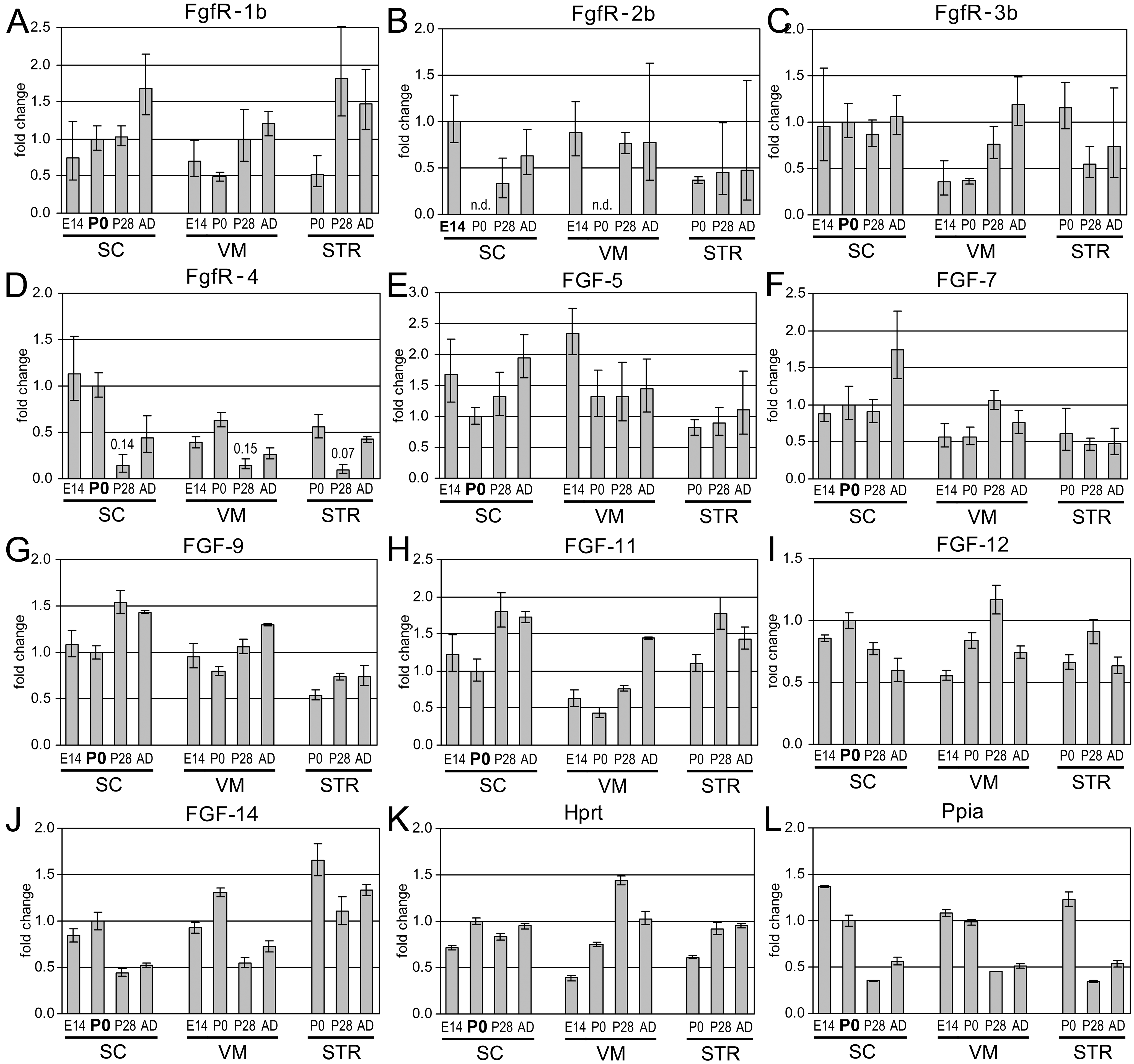

Supplement: Figure S1 — Low expressed FgfR-receptors , stable expressed FGF-ligands and additional control genes. (A–D) Expression of the low expressed FgfR-1b (A), FgfR-2b (B), FgfR-3b (C) and FgfR-4 (D) remained stable (<2 fold changes) throughout development of most tissues analyzed, except for 3 fold increased FgfR-1b in STR (A), 3 fold increased FgfR-3b in VM (C) and temporary decreased expression of FgfR-4 in all 3 tissues at P28 (D). (E–J) Six FGF-ligands FGF-5 (E), FGF-7 (F), FGF-9 (G), FGF-11 (H), FGF-12 (I), FGF-14 (J) remained stable expressed throughout all stages and tissues analyzed. (K,L) Two additional control genes Hprt (K) and Ppia (L) showed minor variation (between 0.3 to 1.4 fold changes) compared to Gapdh used for normalization. Expression of Ppia was consistently 2 fold decreased in stages P28 and AD in all three tissues. Note the different scaling of the y-axis. (TIF) [file pone.0023564.s001.tif]

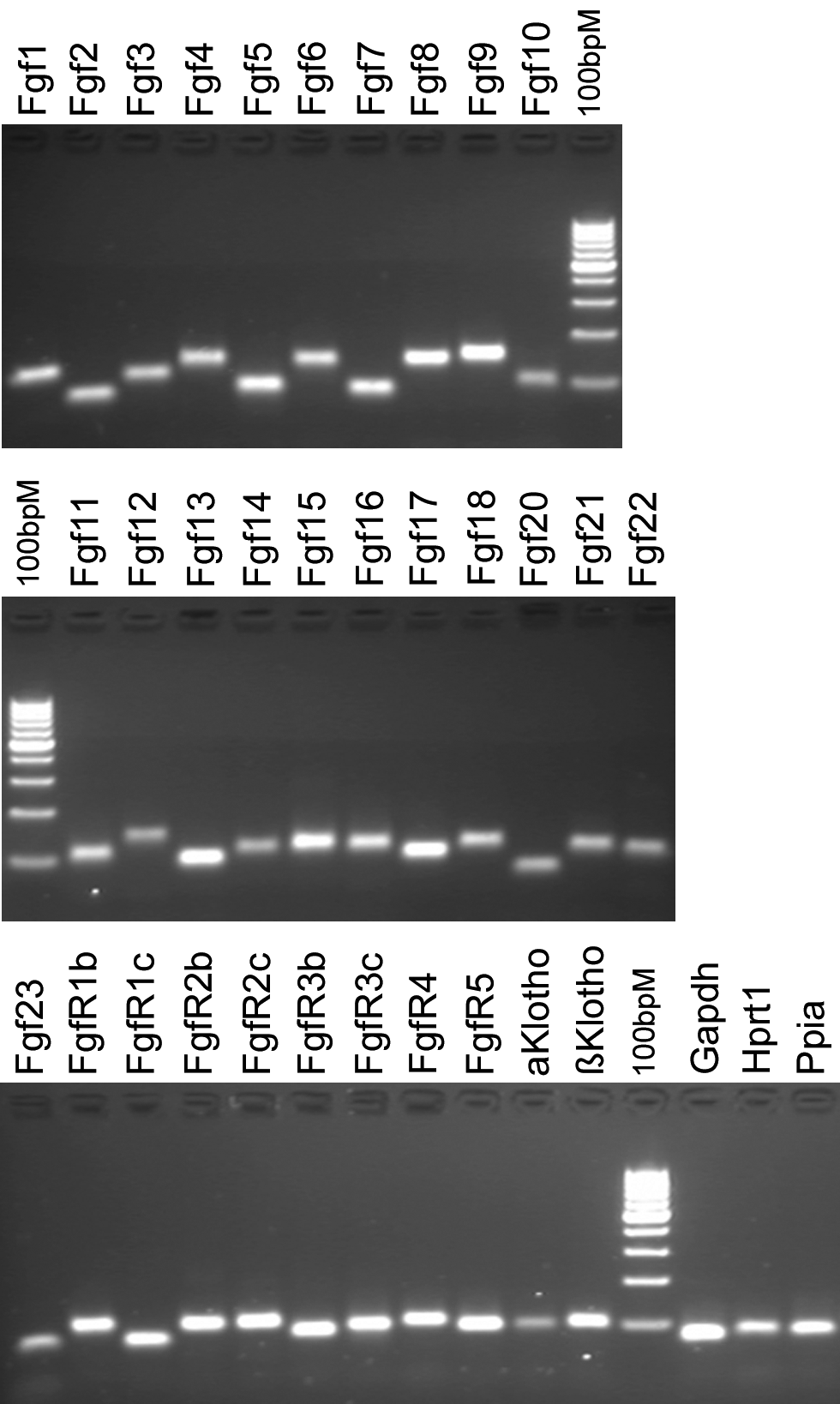

Supplement: Figure S2 — PCR product gel analysis. After qRT-PCR cycling, PCR reactions were separated on a 2% agarose gel together with a 100 bp size marker (100 bp–1 kb in 100 bp steps). All primer pairs produced single PCR-products of the expected size (compare Table S1). (TIF) [file pone.0023564.s002.tif]
