# Supplementary material for: FGF-2 Deficiency Does Not Influence FGF Ligand and Receptor Expression during Development of the Nigrostriatal System
Source: PLoS One. 2011 Aug 18;6(8):e23564. doi: 10.1371/journal.pone.0023564 (PMC3158085; doi:10.1371/journal.pone.0023564)
Supplement: Table S1 — FGF and FgfR primer sequences. Characteristic parameters of qPCR-products (length and melting point) are summarized in the 3rd column. Raw ΔCT values obtained from the reference tissue (P0 SC or exceptions AD SC, P0 VM, E14 SC, P28 SC) are indicated in the 4th column. Abbreviations: aFGF, acidic FGF; bFGF, basic FGF; CT, threshold cycle; FHF, fibroblast growth factor homologous factor; SC, spinal cord; STR, striatum; VM, ventral mesencephalon. (DOC) [file pone.0023564.s003.doc]

**Supplemental Table 1: FGF and FgfR primer sequences.**

| Gene name, synonym (GenBank ID, primer position) | Primer sequences | Product size, melting point | ΔC_T_ value (P0 SC) |
| --- | --- | --- | --- |
| FGF-1, aFGF (NM_010197.3, 441 – 561 bp) | 5´-AAGGGCTTTTATACGGCTCG-3´, 5´-CCCACAAACCAGTTCTTCTCC-3´ | 121 bp, 77.9°C | 8.2 |
| FGF-2, bFGF (NM_008006.2, 354 – 431 bp) | 5´-GAGAAGAGCGACCCACACG-3´, 5´-GGCACACACTCCCTTGATAGA-3´ | 78 bp, 77.2°C | 10.3 |
| FGF-3 (NM_008007.2, 327 – 434 bp) | 5´-GATTACTGCGGTGGAAGTGG-3´, 5´-GCGTTGTAGTGATCCGAAGC-3´ | 108 bp, 81.3°C | 12.2 |
| FGF-4 (NM_010202.5, 437 – 572 bp) | 5´-CACGAGGGACAGTCTTCTGG-3´, 5´-ACACTCGTCGGTAAAGAAAGG-3´ | 136 bp, 84.7°C | 14.8 (AD SC) |
| FGF-5 (NM_010203.4, 528 – 613 bp) | 5´-GATCTACCCGGATGGCAAAG-3´, 5´-CAATCCCCTGAGACACAGCA-3´ | 86 bp, 76.2°C | 12.7 |
| FGF-6 (NM_010204.1, 399 – 533 bp) | 5´-GAGATCTCCACGGTAGAACGG-3´, 5´-GAGGGTTTCTCGGAACTTGC-3´ | 135 bp, 80.5°C | n.d. |
| FGF-7 (NM_008008.4, 691 – 772 bp) | 5´-CCTGAGGATTGACAAACGAGG-3´, 5´-CACGGTCCTGATTTCCATGA-3´ | 82 bp, 76.3°C | 10.8 |
| FGF-8 (NM_010205.2, 584 – 722 bp) | 5´-CATGGCAGAAGACGGAGACC-3´, 5´-GTTGCTCTTGGCAATTAGCTTCC-3´ | 139 bp, 81.0°C | 14.7 |
| FGF-9 (NM_013518.4, 707 – 856 bp) | 5´-GGACTCTACCTCGGCATGAA-3´, 5´-GTATCTCCTTCCGGTGTCCA-3´ | 150 bp, 77.7°C | 6.8 |
| FGF-10 (NM_008002.4, 957 – 1054 bp) | 5´-TCACGATTGAGAAGAACGGC-3´, 5´-GGCAACAACTCCGATTTCC-3´ | 98 bp, 78.2°C | 8.2 |
| FGF-11, FHF-3 (NM_010198.1, 385 – 491 bp) | 5´-GCTGAGGGGCTATTGTACAGC-3´, 5´-TGACGGTAGAGAGCAGAGGC-3´ | 107 bp, 78.5°C | 7.4 |
| FGF-12, FHF-1 (NM_183064.3, 291 – 431 bp) | 5´-GCAGATGCATCCAGATGGTA-3´, 5´-TCTCCATTCATGGCCACATA-3´ | 141 bp, 79.8°C | 4.2 |
| FGF-13, FHF-2 (NM_010200.2, 925 – 1019 bp) | 5´-TGTGAAGAAGAACAAGCCTGC-3´, 5´-AGAACTCCGTGAGATCGTGC-3´ | 95 bp, 79.1°C | 3.6 |
| FGF-14, FHF-4 (NM_010201.4, 556 – 673 bp) | 5´-GGAAGGGCAAGTTATGAAAGG-3´, 5´-TTCACCAACATCATGCAAGG-3´ | 118 bp, 77.1°C | 5.8 |
| FGF-15, FGF-19 (NM_008003.2, 319 – 445 bp) | 5´-GCTGGTCCCTATGTCTCCAAC-3´, 5´-TGATGGCAATCGTCTTCAGAG-3´ | 127 bp, 81.5°C | 10.8 |
| FGF-16 (NM_030614.2, 406 – 530 bp) | 5´-CCTAGGAATGAATGAGCGAGG-3´, 5´-CCGAGTGTTTGTACAAGGTGG-3´ | 125 bp, 77.6°C | 13.1 |
| FGF-17 (NM_008004.4, 784 – 895 bp) | 5´-TACATTCGGCAGCAGAGTCC-3´, 5´-CACGCAGTCTTTGCTCTTCC-3´ | 112 bp, 81.1°C | 11.9 |
| FGF-18 (NM_008005.1, 431 – 561 bp) | 5´-TCAAGGGCAAGGAGACAGAA-3´, 5´-CATCAGGGCCGTGTAGTTGT-3´ | 131 bp, 79.4°C | 6.9 |
| FGF-20 (NM_030610.2, 585 – 667 bp) | 5´-GGACAGTGGCCTGTACCTTG-3´, 5´-CCCTGAAGATGCATTCAGAAGT-3´ | 83 bp, 74.6°C | 13.7 (P0 VM) |
| FGF-21 (NM_020013.4, 422 – 549 bp) | 5´-AGTCTCCTGGAGCTCAAAGCC-3´, 5´-CAGGCCTCAGGATCAAAGTGA-3´ | 128 bp, 80.3°C | n.d. |
| FGF-22 (NM_023304.1, 115 – 236 bp) | 5´-TCCACTCACTTTTTCCTGCG-3´, 5´-GCTTTGATCACCACAGTGCC-3´ | 122 bp, 83.7°C | 11.2 |
| FGF-23 (NM_022657.3, 386 – 463 bp) | 5´-AGGAGCCATGACTCGAAGGTT-3´, 5´-TTCTCTGGGCTGAAGTGAAGC-3´ | 78bp, 77.4°C | n.d. |
| FgfR-1b (AF176552, 696 – 805 bp) | 5´-CTTGCCGTATGTCCAGATCC-3´, 5´-CCTTACACACATACTCCCCGC-3´ | 110 bp, 80.1°C | 12.4 |
| FgfR-1c (NM_010206.2, 1658 – 1734 bp) | 5´-CTTGCCGTATGTCCAGATCC-3´, 5´-TCCGTAGATGAAGCACCTCC-3´ | 77 bp, 76.3°C | 6.2 |
| FgfR-2b (NM_201601.2, 1945 – 2052 bp) | 5´-ACTGTCCTGCCCAAACAGC-3´, 5´-GCAGGCGATTAAGAAGACCC-3´ | 108 bp, 80.4°C | 13.4 (E14 SC) |
| FgfR-2c (NM_010207.2, 2286 – 2394 bp) | 5´-TGCATGGTTGACAGTTCTGC-3´, 5´-GCAGGCGATTAAGAAGACCC-3´ | 109 bp, 78.5°C | 7.4 |
| FgfR-3b (NM_001163217.1, 949 – 1037 bp) | 5´-GAAGCACGTGGAAGTGAACG-3´, 5´-CCACATTCTCACTGATCCAGG-3´ | 89 bp, 81.4°C | 12.0 |
| FgfR-3c (NM_008010.4, 1154 – 1249 bp) | 5´-GAAGCACGTGGAAGTGAACG-3´, 5´-TCCTTGTCGGTGGTGTTAGC-3´ | 96 bp, 82.8°C | 6.4 |
| FgfR-4 (NM_008011.2, 693 – 796 bp) | 5´-TACCATCCACTGGCTCAAGG-3´, 5´-CCACACTTTCCATCACCAGG-3´ | 104 bp, 81.6°C | 11.5 |
| FgfRl1, FgfR-5 (NM_054071.2, 1467 – 1559 bp) | 5´-GGTGCAAATACCATGGGCTAC-3´, 5´-CGATGAAGAAGCCATAGGAGG-3´ | 93 bp, 79.0°C | 7.6 |
| α-Klotho, Kl (NM_013823.2, 1672 – 1764 bp) | 5´-TTCCCTGTGACTTTGCTTGG-3´, 5´-CCCACAGATAGACATTCGGG-3´ | 93 bp, 77.0°C | 10.1 |
| β-Klotho, Klb (NM_031180.2, 1278 – 1375 bp) | 5´-AGGACACCACGGCCATCTAC-3´, 5´-CCAGGCCGTATAACCAAACAC-3´ | 98 bp, 75.7°C | 13.5 (P28 SC) |
| Gapdh (NM_008084.2, 656 – 733 bp) | 5´-GAACATCATCCCTGCATCCA-3´, 5´-CCAGTGAGCTTCCCGTTCA-3´ | 78 bp, 81.1°C | 0 |
| Hprt (NM_013556.2, 256 – 342 bp) | 5´-TTCCTCATGGACTGATTATGGACA-3´, 5´-AGAGGGCCACAATGTGATGG-3´ | 87 bp, 77.2°C | 4.4 |
| Ppia (NM_008907.1, 384 – 468 bp) | 5´-TGCACTGCCAAGACTGAATG-3´, 5´-CCATGGCTTCCACAATGTTC-3´ | 85 bp, 78.1°C | -0.3 |

Characteristic parameters of qPCR-products (length and melting point) are summarized in the 3^rd^ column. Raw ΔC_T_ values obtained from the reference tissue (P0 SC or exceptions AD SC, P0 VM, E14 SC, P28 SC) are indicated in the 4^th^ column. Abbreviations: aFGF, acidic FGF; bFGF, basic FGF; C_T_, threshold cycle; FHF, fibroblast growth factor homologous factor; SC, spinal cord; STR, striatum; VM, ventral mesencephalon.
